# Extrinsic Parameter Calibration Method for a Visual/Inertial Integrated System with a Predefined Mechanical Interface

**DOI:** 10.3390/s19143086

**Published:** 2019-07-12

**Authors:** Chenguang Ouyang, Shuai Shi, Zheng You, Kaichun Zhao

**Affiliations:** 1 State Key Laboratory of Precision Measurement Technology and Instruments, Tsinghua University, Beijing 100084, China; 2 Astronaut Center of China, Beijing 100094, China

**Keywords:** visual/inertial integrated system, extrinsic parameter calibration, corner detection, motion blur

## Abstract

For a visual/inertial integrated system, the calibration of extrinsic parameters plays a crucial role in ensuring accurate navigation and measurement. In this work, a novel extrinsic parameter calibration method is developed based on the geometrical constraints in the object space and is implemented by manual swing. The camera and IMU frames are aligned to the system body frame, which is predefined by the mechanical interface. With a swinging motion, the fixed checkerboard provides constraints for calibrating the extrinsic parameters of the camera, whereas angular velocity and acceleration provides constraints for calibrating the extrinsic parameters of the IMU. We exploit the complementary nature of both the camera and IMU, of which the latter assists in the checkerboard corner detection and correction while the former suppresses the effects of IMU drift. The results of the calibration experiment reveal that the extrinsic parameter accuracy reaches 0.04° for each Euler angle and 0.15 mm for each position vector component (1σ).

## 1. Introduction

Visual/inertial integrated systems have been used across many contexts, including indoor [1,2,3], underwater [4], space environments [5], and taking some measurement tasks [6,7]. To achieve accurate navigation and measurement, camera and inertial sensor frames should be aligned to the carrier frame in a process often called extrinsic parameter calibration or alignment. In this process, a coordinate transforming relationship is established between the sensor frame and the system body frame. Without calibrating the extrinsic parameters, the navigation errors will be coupled with the misalignment errors [8].

Previous studies have developed theoretical models of the camera [9] and the inertial measurement unit (IMU) [10,11,12], based on which the extrinsic parameters of visual/inertial integrated systems have been calibrated. These parameters are generally calibrated in three ways. First, these parameters are calibrated based on the corresponding rotation differences. Given that both the camera and IMU can evaluate their own rotations, a calibration method that minimizes the overall matching error of the rotation matrix between the visual and inertial coordinate systems in multiple rotations has been proposed in [13], whereas You et al. [14] developed a calibration method that relies on the angular velocity differences between the IMU and camera in the subsequent rotations. Second, these parameters are calibrated based on the vertical direction constraint. Given that the gravity and vertical line are measured by an IMU and camera in the same direction (i.e., vertical direction), a calibration method that uses this direction as reference has been proposed in [15,16,17]. Third, these parameters are calibrated based on filtering or optimization. In vision-aided inertial navigation, the coordinate transforming relation between the IMU and camera is estimated. The methods developed by Mourikis et al. [18] and Kelly and Sukhatme [19] estimate the extrinsic parameters based on the extended Kalman filter (EKF) and the unscented Kalman filter, respectively. In addition, Kaminer et al. [20] proposed a method based on nonlinear, globally stable filters, while Yang and Shen [21] proposed an optimization-based calibration method.

Most of these methods align the camera frame to the IMU frame. However, in some cases where a high-precision operation is required, the visual/inertial system should be aligned to the system body frame and not to the IMU frame. In addition, when a carrier, such as a spacecraft, is too heavy to conduct a calibration operation, the before mentioned methods cannot fully solve the problem. Previous studies have attempted to address this problem by aligning the sensors frame to the system body frame. For instance, Wendel and Underwood [22] aligned the line scanning cameras to the ground vehicle frame and achieved an accuracy of 0.06 m in translation and 1.05° in rotation, while Shi et al. [23] aligned three cameras to the body system frame and achieved 0.6 mm and 0.1° translation and rotation accuracies, respectively. Only the camera frame is aligned to the body frame and the accuracy can be further improved. Given that self-integrated and some commercial IMUs lack a mechanical interface. Foxlin and Naimark [24] aligned the IMU to the system body frame defined by a mechanical interface by rolling on a flat surface and then aligned the camera to the system body frame by using a calibration device. However, the accuracy of the extrinsic parameters is not clearly shown. Pittelkau [25] proposed a method for aligning an inertial sensor assembly of three fiber-optic gyros, two star trackers, and a camera to a spacecraft by using an alignment Kalman filter, but this method has only been validated via simulation.

We present an extrinsic parameter calibration method that uses IMU and camera measurements to align the IMU and camera frames to the system body frame defined by the mechanical interface. This method offers two advantages. First, the sensor frames are aligned to the carrier frame through the mechanical interface without requiring a complex calibration process. Second, this method can be seen as a standard calibration step for factory production and user operation. The operation process of calibration is not complex. Fix a checkerboard on the wall within the field of view of the camera, fix the integrated system on a two-axis turntable, and then manually control this turntable to swing around its two axes. The checkerboard is in the camera’s field of view all the time.

Our novelties are that,

1. In this study, we exploit the complementary nature of IMU and camera to improve the calibration accuracy. The camera measurements can suppress the inertial propagation drift, whereas the motion parameters evaluated by IMU can accurately extract the feature points under a smearing effect. We exploit the complementary nature of these two components to improve the calibration accuracy.
2. The method for IMU aided checkerboard corner correction under motion blur is introduced, which is the tip step to improve accuracy of camera calibration. We use the IMU to evaluate the motion parameters, and use these motion parameters to eliminate the effects of motion blur in image. It is described in Section 2.3. The extrinsic parameters are evaluated based on EKF, and the rotation angle is evaluated using camera measurement to suppress IMU’s drift.
3. The calibration process can be a standard calibration step for factory production and user operation. The turntable is the standard equipment for IMU calibration, and the checkerboard is the standard device for camera calibration. So, the cost is low, and the calibration process is simple and convenient. Our experiment results indicate that our proposed method is valid and achieves a fair level of accuracy. Our method can also align the camera frame to the IMU frame.

The rest of this paper is organized as follows. Section 2 introduces the mathematical model and the proposed calibration method. Section 3 presents the simulation and the real-world experiment. Section 4 concludes the paper.

## 2. Calibration Method

**Figure 1:**
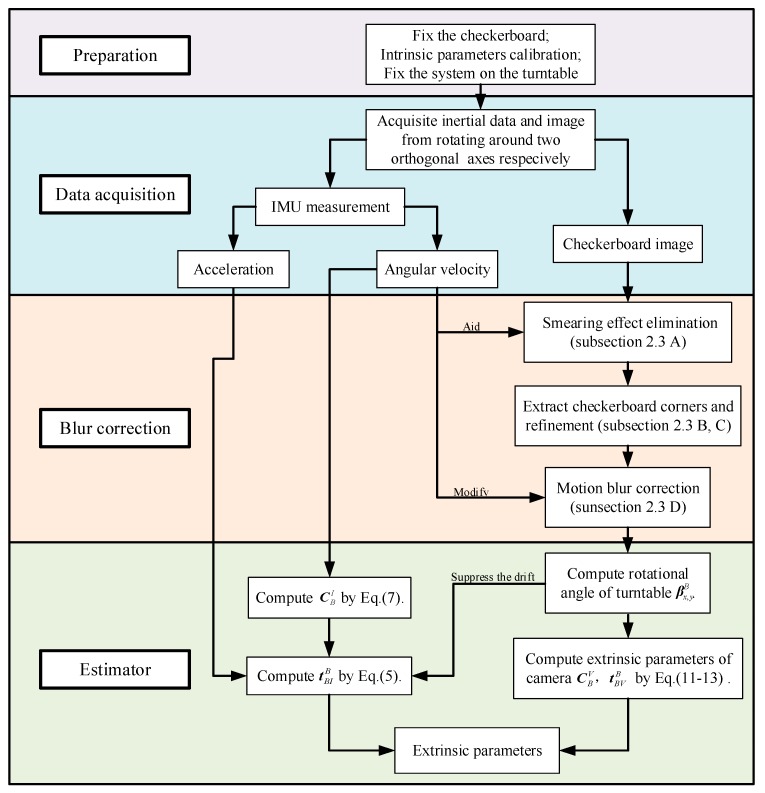
Extrinsic calibration process.

The main idea of the proposed calibration method is shown in Figure 1, which can help understand the part of the calibration. There are four step of this calibration. Firstly, “Preparation”, the following preparations should be performed before the system calibration:

The checkerboard should be fixed in an appropriate location that can be observed by the cameras while the system rotates along with the turntable;
The intrinsic parameters of the camera and IMU should be calibrated;
The visual/inertial system should be fixed on the turntable.

Secondly, “data acquisition”, the turntable is controlled manually to rotate around two orthogonal axes, such as the *x* and *y* axes, for the checkerboard image acquisition by camera and the inertial data acquisition by IMU, respectively. 

Thirdly, “blur correction”, the motion blur exits in the checkerboard image. The checkerboard corners are detected with the aid of IMU to achieve higher accuracy. 

Finally, “estimator”, we estimate the extrinsic parameters based on EKF.

The main idea of this calibration method is that establish the equations that provide the extrinsic parameters and solve these equations. To better understand the method, the basic measurement models of the IMU and camera are described in the Section 2.1. Because the calibration operation process is swinging motion, the detailed measurement equations of IMU and camera under swinging motion are described in Section 2.2. We exploit the complementary nature of IMU and camera to improve the calibration accuracy, the method of inertial aided checkerboard corner extraction under motion blur is described in Section 2.3. And the extrinsic parameters are evaluated based on EKF, which is shown in Section 2.4. The rotation angle of turntable is evaluated by camera measurement to suppress the IMU’s drift. 

### 2.1. Measurement Model

The measurement model for IMU is formulated as follows (The superscripts and subscripts *F*, *B*, *I*, and *V* represent the frame of checkerboard, system body, IMU, and camera, respectively, while *F*_0_, *B*_0_, *I*_0_, and *V*_0_ represent the frame fixed on the earth and coincident to the frame of checkerboard, system body, IMU, and camera in its initial pose, respectively).

(1) $$\begin{array}{l} f_{meas}^{I}=a_{true}^{I}-g_{}^{I}+b_{a}+n_{a}\\ \omega_{meas}^{I}=\omega_{true}^{I}+b_{g}+n_{g} \end{array},$$

where $a_{true}^{I}$ is the true specific force excluding gravity, $\omega_{true}^{I}$ is the true angular velocity, $g_{}^{I}$ is the gravity expressed in the IMU frame, $b_{a}$ and $b_{g}$ are the bias errors of the accelerometer and gyro, respectively, and $n_{a}$ and $n_{g}$ are the process white Gaussian noises of the accelerometer and gyro, respectively. Both $b_{a}$ and $b_{g}$ show minimal changes within a short period.

We use the ideal pinhole model as the measurement model for the camera that maps a 3D point $P^{F}\left(x^{F},y^{F},z^{F}\right)$ to a 2D image p(u,v) as [26]

(2) $$z_{c}\left[\begin{array}{c} u\\ v\\ 1 \end{array}\right]=\left[\begin{array}{cccc} \alpha_{x} & \gamma & u_{0} & 0\\ 0 & \alpha_{y} & v_{0} & 0\\ 0 & 0 & 1 & 0 \end{array}\right]\left[\begin{array}{cc} C_{F}^{V} & t_{FV}^{V}\\ 0 & 1 \end{array}\right]\left[\begin{array}{c} P_{}^{F}\\ 1 \end{array}\right]=\left[\begin{array}{cc} M & 0 \end{array}\right]\left[\begin{array}{cc} C_{F}^{V} & t_{FV}^{V}\\ 0 & 1 \end{array}\right]\left[\begin{array}{c} P_{}^{F}\\ 1 \end{array}\right],$$

where $z_{c}$ is the camera frame optic axis coordinate of point P, $\alpha_{x}$, and $\alpha_{y}$ are the scale factors of the u and v axes of the image plane, respectively, γ is the non-orthogonal factor of the image plane axes, $\left(u_{0}^{},v_{0}^{}\right)$ is the pixel coordinate of the camera principal point, $C_{F}^{V}$ is the 3 × 3 rotation matrix, $t_{FV}^{V}$ is the 3D translation vector, and M is the intrinsic parameter matrix. In the actual situation, the principal point, focal length deviation, distortion, and other error factors should be considered based on the ideal camera model. These parameters can be acquired via an intrinsic calibration, such as by employing Zhang’s method [27].

### 2.2. Extrinsic Parameter Calibration Method for a Visual/Inertial Integrated System

The physical quantities that should be calibrated include the following:

1. The translation matrices $C_{B}^{V}$ and $C_{B}^{I}$, which occur between the system body frame and the camera frame as well as between the system body frame and the IMU frame, respectively.
2. The position vectors $t_{BV}^{B}$ and $t_{BI}^{B}$, which are derived from the camera and IMU principal points to the origin of the system body frame, respectively.

Given that IMU can measure angular velocity and acceleration vectors during rotation, the constraint equations of inertial vectors and the extrinsic parameters of IMU can be established. When the turntable rotates around by one axis, the position vector and Euler angle along the rotation axis cannot be computed. However, when the turntable rotates by around two orthogonal axes, extrinsic parameters of IMU can be computed. Fortunately, IMU calibration is generally performed by using a turntable with two or three orthogonal axes.

Camera calibration generally involves the use of a checkerboard. In the calibration process, both the intrinsic parameters and the translation matrix and position vector between the camera and checkerboard frames can be computed. By turning the turntable, the constraint equation can be established based on the relationship between the extrinsic parameters of camera and the change in the camera position and attitude. The constraint equations of the visual/inertial system are described as follows.

In general, when the Coriolis acceleration is ignored during rotation, the accelerometer measurement is

(3) $$\begin{array}{c} f_{}^{I}=a_{n}^{I}+a_{\tau }^{I}+g_{}^{I}+b_{a}^{}\\ \;\;\;\;\;\;\;\;\;\;\;\;\;\;\;\;=C_{B}^{I}\left(a_{n}^{B}+a_{\tau }^{B}+g_{}^{B}\right)+b_{a}^{} \end{array},$$

where $a_{n}^{B}={\left[\Omega_{}^{B}\right]}^{2}t_{BI}^{B}$ is the radial acceleration, $a_{\tau }^{B}=E_{}^{B}t_{BI}^{B}$ is the tangential acceleration, $g_{}^{I}$ is the gravitational acceleration expressed in the IMU frame, and $C_{B}^{I}$ is the translation matrix from the system body frame to the IMU frame, which can be calculated by the Euler angle $\psi_{B}^{I}$.

$\Omega_{}^{B}$ and $E_{}^{B}$ are skew-symmetric matrices defined as

(4) $$\Omega_{}^{B}=\left[\omega_{}^{B}\times \right]=\left[\begin{array}{ccc} 0 & -\omega_{z}^{} & \omega_{y}^{}\\ \omega_{z}^{} & 0 & -\omega_{x}^{}\\ -\omega_{y}^{} & \omega_{x}^{} & 0 \end{array}\right],\;E_{}^{B}=\left[\varepsilon_{}^{B}\times \right]=\left[\begin{array}{ccc} 0 & -\varepsilon_{z}^{} & \varepsilon_{y}^{}\\ \varepsilon_{z}^{} & 0 & -\varepsilon_{x}^{}\\ -\varepsilon_{y}^{} & \varepsilon_{x}^{} & 0 \end{array}\right],$$

where $\omega_{}^{B}={\left[\begin{array}{ccc} \omega_{x} & \omega_{y} & \omega_{z} \end{array}\right]}^{T}$ is the angular velocity vector, and $\varepsilon_{}^{B}={\left[\begin{array}{ccc} \varepsilon_{x} & \varepsilon_{y} & \varepsilon_{z} \end{array}\right]}^{T}$ is the angular acceleration vector.

Therefore, the accelerometer measurement can be rewritten as

(5) $$\begin{array}{ll} f^{I} & =C_{B}^{I}\left(a_{n}^{B}+a_{\tau }^{B}+g_{}^{B}\right)+b_{a}^{}\\ & =C_{B}^{I}C_{B_{0}}^{B}\left[\left({\left(\Omega_{}^{B_{0}}\right)}^{2}+E_{}^{B_{0}}\right)t_{B_{0}I_{0}}^{B_{0}}+C_{I_{0}}^{B_{0}}g_{}^{I_{0}}\right]+b_{a}\\ & =C_{B}^{I}C_{B_{0}}^{B}\left[\left({\left(\Omega_{}^{B_{0}}\right)}^{2}+E_{}^{B_{0}}\right)t_{BI}^{B}+C_{I}^{B}g_{}^{I_{0}}\right]+b_{a} \end{array},$$

where $g_{}^{I_{0}}$ is the gravity accelerometer expressed in the {$I_{0}$} frame and can be written as

(6) $$g_{}^{I_{0}}=f_{}^{I_{0}}-b_{a}^{},$$

where $f_{}^{I_{0}}$ is the accelerometer measurement in the initial pose. Therefore, $b_{a}^{}$ must be initially evaluated to determine $g_{}^{I_{0}}$. The gyro measurement is formulated as

(7) $$\omega_{}^{I}=C_{B}^{I}\omega_{}^{B}=C_{B}^{I}C_{B_{0}}^{B}\omega_{}^{B_{0}}.$$

An integrated system rotates with a two-axis turntable. When turntable rotates around the *x* axis, we have

(8) $$C_{B_{0}}^{B}=\left[\begin{array}{ccc} 1 & & \\ & \cos\beta_{x} & \sin\beta_{x}\\ & -\sin\beta_{x} & \cos\beta_{x} \end{array}\right],\;f_{x}^{I}=f_{}^{I},\;\omega_{x}^{I}=\omega_{}^{I},$$

where $\beta_{x}$ is the rotation angle of the turntable when rotating around the *x* axis.

However, when rotating around the *y* axis, we have

(9) $$C_{B_{0}}^{B}=\left[\begin{array}{ccc} \cos\beta_{y} & & \sin\beta_{y}\\ & 1 & \\ -\sin\beta_{y} & & \cos\beta_{y} \end{array}\right],\;f_{y}^{I}=f_{}^{I},\;\omega_{y}^{I}=\omega_{}^{I},$$

where $\beta_{y}$ is the rotation angle of the turntable when rotating around the y axis.

$\beta_{k}^{}$, k=x,y, can be computed by integrating the gyro measurement, but an accumulation error is observed. This error is then evaluated based on the camera measurement in the Kalman process.

The camera measurement can be formulated as

(10) $$\begin{array}{ll} z & ={\left[\begin{array}{cccc} u_{x}^{} & v_{x}^{} & u_{y}^{} & v_{y}^{} \end{array}\right]}^{T}\\ \left[\begin{array}{ll} u_{x}^{} & v_{x}^{} \end{array}\right] & =\left[\begin{array}{llllll} u_{x,1} & \cdots & u_{x,n} & v_{x,1} & \cdots & v_{x,n} \end{array}\right]\\ \left[\begin{array}{cc} u_{y}^{} & v_{y}^{} \end{array}\right] & =\left[\begin{array}{cccccc} u_{y,1} & \cdots & u_{y,n} & v_{y,1} & \cdots & v_{y,n} \end{array}\right] \end{array},$$

where subscripts *x* and *y* indicate that the turntable rotates around the *x* and *y* axes, respectively, $\left(u_{i}^{},v_{i}^{}\right)$ is the pixel coordinate of each checkerboard corner, and *n* is the number of checkerboard corners.

The value of $\left(u_{i}^{},v_{i}^{}\right)$ can be computed as

(11) $$\begin{array}{l} \left[\begin{array}{c} u_{i}^{}\\ v_{i}^{} \end{array}\right]=z_{i}^{}\left[\begin{array}{c} x_{i}^{}\\ y_{i}^{} \end{array}\right],\;\mathrm{where}\left[\begin{array}{c} x_{i}^{}\\ y_{i}^{}\\ z_{i}^{} \end{array}\right]=M\left[\begin{array}{c} x_{i}^{V}\\ y_{i}^{V}\\ z_{i}^{V} \end{array}\right],\left[\begin{array}{c} x_{i}^{V}\\ y_{i}^{V}\\ z_{i}^{V} \end{array}\right]=P_{i}^{V}=C_{V_{0}^{}}^{V_{}^{}}P_{i}^{V_{0}^{}}-t_{V_{0}^{}V_{}^{}}^{V_{}^{}} \end{array},$$

$\left(u_{i}^{V},v_{i}^{V}\right)$ can be defined as

(12) $$\left[\begin{array}{c} u_{i}^{V}\\ v_{i}^{V} \end{array}\right]=z_{i}^{V}\left[\begin{array}{c} x_{i}^{V}\\ y_{i}^{V} \end{array}\right],$$

and $P_{i}^{V_{0}^{}}$ and $t_{V_{0}^{}V}^{V}$ in Equation (11) can be written as

(13) $$\begin{array}{ll} P_{i}^{V_{0}^{}} & =C_{F}^{V_{0}^{}}P_{i}^{F}+t_{V_{0}^{}F}^{V_{0}^{}}\\ t_{V_{0}^{}V_{}^{}}^{V_{}^{}} & =C_{B}^{V}\left(t_{BV}^{B}-C_{B_{0}}^{B}t_{BV}^{B}\right) \end{array},$$

where $P_{i}^{F}$ is the 3D coordinate of the *i*th checkerboard corner in the checkerboard frame. In Equations (8) and (9), $C_{B_{0}}^{B}$ can be computed based on the rotational angle of the turntable and correspond to the rotation of the turntable around the *x* and *y* axes, respectively. 

Meanwhile, $P_{i}^{F}$ is determined by the user, and $\left(u_{i}^{},v_{i}^{}\right)$ denotes the pixel coordinate of the checkerboard corner in an image. If the number of checkerboard corners is >8, then $C_{F}^{V_{0}^{}}$ and $t_{V_{0}^{}F}^{V_{0}^{}}$ can be evaluated by using least squares.

### 2.3. Checkerboard Corner Extraction Method under Motion Blur

During the working process of a digital camera, the shutter needs to open for a moment to project light onto the photographic material. This brief moment is called the exposure time. Under highly dynamic conditions, the relative pose between the camera and the object changes evidently during the exposure time, thereby blurring or stretching the generated image [28]. The calibration of the extrinsic parameters requires a rotation, especially for IMU. Therefore, for the camera measurements, we must extract the checkerboard corners during rotation, but a motion blur may be generated in the process. Conventional checkerboard corner detection methods compute the local optimum value [29] with static images. Therefore, when noise and motion blur are present in an image, the errors in the extraction results evidently increase. 

We utilize inertial data to eliminate the smearing effect and then extract the checkerboard corners with the aid of the IMU data. The corner detection algorithm is developed as follows. The Lucy–Richardson method is used to rectify the blurred image in step A, and the conventional corner detection method is used to find the corners’ pixel coordinates in step B. Given that checkerboard corners are constrained on a few lines, we add a linear constraint to refine the checkerboard corner location. Through the linear constraint, the corners’ pixel coordinates are refined in step C. Finally, IMU measurement is used to correct the position of corner detected for movement in step D.

- A. Pretreatment and smearing effect elimination.

We deblur the image using the Lucy–Richardson method [30,31,32]. The point–spread function (PSF) is a 2D Gaussian model $N\left(0,0,\sigma_{x}^{2},\sigma_{y}^{2}\right)$, and $\sigma_{x}^{}$ and $\sigma_{y}^{}$ denote the standard deviations in the *x* and *y* axes, respectively. If the velocity of the checkerboard expressed in the camera frame coincides with the x axis, then a motion blur is only observed in the *x* axis. Therefore, the PSF can be written as $N\left(0,0,\sigma_{}^{2},0\right)$, and the corresponding covariance matrix is

(14) $$C=\left[\begin{array}{cc} \sigma^{2} & \\ & 0 \end{array}\right],$$

where $\sigma^{2}=\eta \omega_{}^{B}$, η is the scale factor, and $\omega_{}^{B}$ is the angular velocity of rotation. 

When the cross angle between the velocity of the checkerboard is expressed in the camera frame and the x axis of the camera frame is θ, the covariance matrix of PSF can be written as

(15) $$C_{\theta }^{}=R\left(\theta \right)CR{\left(\theta \right)}^{T},$$

where $R\left(\theta \right)=\left[\begin{array}{cc} \cos\theta & \sin\theta \\ -\sin\theta & \cos\theta \end{array}\right]$. θ can be evaluated by camera data. 

**Figure 2:**
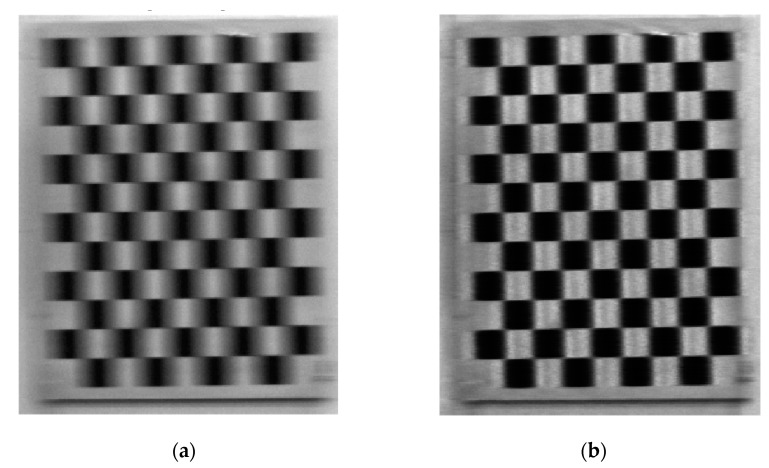
Image deblurring using the Lucy–Richardson method. (**a**) Original and (**b**) deblurred images.

Figure 2 compares the original and deblurred images and shows that the motion blur has been effectively eliminated.

- B. Corner detection.

This step is the same as that in the conventional method. The corners in the image, including the complete checkerboard, are examined. $I_{x}$ and $I_{y}$ denote the *x* (horizontal) and *y* (vertical) components of the 2D numerical gradient, and the first and second derivatives are computed as

(16) $$\begin{array}{c} I_{x}=\frac{\partial I}{\partial x},\;I_{y}=\frac{\partial I}{\partial y},\;I_{xy}=\frac{\partial I}{\partial x\partial y}\\ I_{45}=I_{x}\cos\left(\frac{\pi }{4}\right)+I_{y}\sin\left(\frac{\pi }{4}\right),\;I_{-45}=I_{x}\cos\left(-\frac{\pi }{4}\right)+I_{y}\sin\left(-\frac{\pi }{4}\right)\\ I_{45,x}=\frac{\partial I_{45}}{\partial x},\;I_{45,y}=\frac{\partial I_{45}}{\partial y},\;I_{45,45}=I_{45,x}\cos\left(-\frac{\pi }{4}\right)+I_{45,y}\sin\left(-\frac{\pi }{4}\right) \end{array}.$$

The gradient direction is evaluated by

(17) $$C_{xy}=\left|I_{xy}\right|-\mu \left(\left|I_{45}\right|+\left|I_{-45}\right|\right),\;C_{45}=\left|I_{45,45}\right|-\mu \left(\left|I_{x}\right|+\left|I_{y}\right|\right),$$

where μ is the weight coefficient.

**Figure 3:**
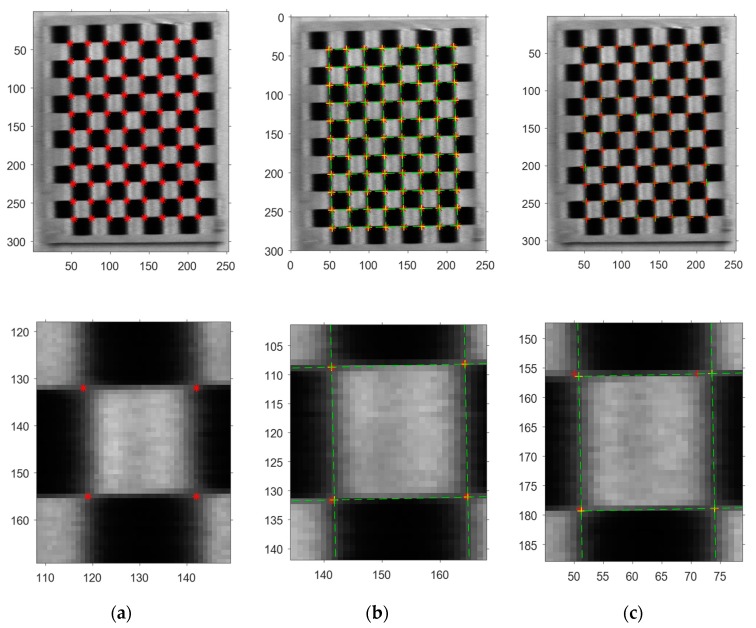
Image process. The pictures in the bottom row show the details of the pictures presented in the upper row. (**a**) Roughly detected checkerboard corners (refer to the red “※”); (**b**) refined results with a linear constraint (refer to the yellow “+”); and (**c**) results after (refer to the yellow “+”) and before refinement (the red “+”).

$C_{xy}$ and $C_{45}$ are computed for each pixel in the picture. Afterward, $C_{xy}$ and $C_{45}$ are determined—one of which is valid—by minimizing the energy function proposed in [29]. Based on the threshold set in advance and non-maxima suppression, the detection accuracy reaches the pixel level. The results are presented in Figure 3a.

- C. Linear constraint refinement.

We can evaluate the lines of checkerboard based on the corners by using least squares. We assume that the line equation is $a_{i}x+b_{j}y+c_{ij}=0$ and that *i* and *j* represent the row and column numbers, respectively. 

The cross points of these lines are then computed for the global optimization of the checkerboard corners (Figure 3b; refer to the yellow ”+”). Figure 3c compares the results before and after refinement, respectively. The error of some corners detected before the refinement has remarkably increased due to motion blur.

- D. IMU-aided checkerboard corner modification

At time *t*, camera and IMU start collecting data simultaneously. The corners extracted have some delay due to the motion blur in the image, with real data at time t+τ/2. τ is the exposure time of camera. We can modify the corner coordinates with the inertial data. The details are presented as follows.

We consider the motion of camera in Δt, and point ***P*** in the camera frame can be written as

(18) $$P_{t}^{V_{t}}=C_{F}^{V_{t}}\left(P_{t}^{F}+t_{FV_{t}}^{F}\right)\;\mathrm{and}$$

(19) $$\begin{array}{ll} P_{t+\Delta t}^{V_{t+\Delta t}} & =C_{F}^{V_{t+\Delta t}}\left(P_{t}^{F}+t_{FV_{t+\Delta t}}^{F}\right)\\ & =C_{V_{t}}^{V_{t+\Delta t}}C_{F}^{V_{t}}\left(P_{t}^{F}+t_{FV_{t}}^{F}+t_{V_{t}V_{t+\Delta t}}^{F}\right)\\ & =C_{V_{t}}^{V_{t+\Delta t}}P_{t}^{V_{t}}+C_{V_{t}}^{V_{t+\Delta t}}t_{V_{t}V_{t+\Delta t}}^{V_{t}} \end{array},$$

where

(20) $$\begin{array}{l} C_{V_{t}}^{V_{t+\Delta t}}=I-\Omega_{t}^{B}\Delta t=\left[\begin{array}{ccc} 1 & \omega_{z}^{t}\Delta t & -\omega_{y}^{t}\Delta t\\ -\omega_{z}^{t}\Delta t & 1 & \omega_{x}^{t}\Delta t\\ \omega_{y}^{t}\Delta t & -\omega_{y}^{t}\Delta t & 1 \end{array}\right]\\ t_{V_{t}V_{t+\Delta t}}^{V_{t}}=t_{V_{t}R_{t}}^{V_{t}}-C_{V_{t+\Delta t}}^{V_{t}}t_{V_{t+\Delta t}R_{t}}^{V_{t+\Delta t}}=\left(I-C_{V_{t+\Delta t}}^{V_{t}}\right)t_{V_{t}R_{t}}^{V_{t}}=-\Omega_{t}^{B}t_{V_{t}R_{t}}^{V_{t}}\Delta t=-\Omega_{t}^{B}t_{VR}^{V}\Delta t \end{array},$$

where $\Omega_{t}^{B}$ is $\Omega_{}^{B}=\left[\omega_{}^{B}\times \right]$ at time *t*.

Equation (19) can be written as

(21) $$\begin{array}{ll} P_{t+\Delta t}^{V_{t}} & =\left(I-\Omega_{t}^{B}\Delta t\right)P_{t}^{V_{t}}-\left(I-\Omega_{t}^{B}\Delta t\right)\Omega_{t}^{B}t_{V_{t}R_{t}}^{V_{t}}\Delta t\\ & \approx \left(I-\Omega_{t}^{B}\Delta t\right)P_{t}^{V_{t}}-\Omega_{t}^{B}t_{VR}^{V}\Delta t \end{array}.$$

$t_{VR}^{V}={\left[\begin{array}{ccc} t_{x}^{} & t_{y}^{} & t_{z}^{} \end{array}\right]}^{T}$ is defined, and Equation (21) is expanded as

(22) $$P_{t+\Delta t}^{V_{t}}=\left[\begin{array}{ccc} 1 & \omega_{z}^{t}\Delta t & -\omega_{y}^{t}\Delta t\\ -\omega_{z}^{t}\Delta t & 1 & \omega_{x}^{t}\Delta t\\ \omega_{y}^{t}\Delta t & -\omega_{x}^{t}\Delta t & 1 \end{array}\right]\left[\begin{array}{c} x_{t}^{V_{t}}\\ y_{t}^{V_{t}}\\ z_{t}^{V_{t}} \end{array}\right]+\left[\begin{array}{ccc} 0 & \omega_{z}^{t}\Delta t & -\omega_{y}^{t}\Delta t\\ -\omega_{z}^{t}\Delta t & 0 & \omega_{x}^{t}\Delta t\\ \omega_{y}^{t}\Delta t & -\omega_{x}^{t}\Delta t & 0 \end{array}\right]\left[\begin{array}{c} t_{x}\\ t_{y}\\ t_{z} \end{array}\right].$$

By substituting Equation (22) into Equation (12), we have

(23) $$\begin{array}{ll} \left[\begin{array}{c} u_{t+\Delta t}^{V}\\ v_{t+\Delta t}^{V} \end{array}\right] & =\left[\begin{array}{c} \frac{x_{t}^{V_{t}}+y_{t}^{V_{t}}\omega_{z}^{t}\Delta t-z_{t}^{V_{t}}\omega_{y}^{t}\Delta t+t_{y}\omega_{z}^{t}\Delta t-\omega_{y}^{t}t_{z}\Delta t}{x_{t}^{V_{t}}\omega_{y}^{t}\Delta t-y_{t}^{V_{t}}\omega_{x}^{t}\Delta t+z_{t}^{V_{t}}+t_{x}\omega_{y}^{t}\Delta t-t_{y}\omega_{x}^{t}\Delta t}\\ \frac{-x_{t}^{V_{t}}\omega_{z}^{t}\Delta t+y_{t}^{V_{t}}+z_{t}^{V_{t}}\omega_{x}^{t}\Delta t-t_{x}\omega_{z}^{t}\Delta t+t_{z}\omega_{x}^{t}\Delta t}{x_{t}^{V_{t}}\omega_{y}^{t}\Delta t-y_{t}^{V_{t}}\omega_{x}^{t}\Delta t+z_{t}^{V_{t}}+t_{x}\omega_{y}^{t}\Delta t-t_{y}\omega_{x}^{t}\Delta t} \end{array}\right]\\ & =\left[\begin{array}{c} \frac{u_{t}^{V}+v_{t}^{V}\omega_{z}^{t}\Delta t-\omega_{y}^{t}\Delta t+\left(t_{y}\omega_{z}^{t}\Delta t-\omega_{y}^{t}t_{z}\Delta t\right)/z_{t}^{V_{t}}}{u_{t}^{V}\omega_{y}^{t}\Delta t-v_{t}^{V}\omega_{x}^{t}\Delta t+\left(t_{x}\omega_{y}^{t}\Delta t-t_{y}\omega_{x}^{t}\Delta t\right)/z_{t}^{V_{t}}+1}\\ \frac{-u_{t}^{V}\omega_{z}^{t}\Delta t+v_{t}^{V}+\omega_{x}^{t}\Delta t+\left(-t_{x}\omega_{z}^{t}\Delta t+t_{z}\omega_{x}^{t}\Delta t\right)/z_{t}^{V_{t}}}{u_{t}^{V}\omega_{y}^{t}\Delta t-v_{t}^{V}\omega_{x}^{t}\Delta t+\left(t_{x}\omega_{y}^{t}\Delta t-t_{y}\omega_{x}^{t}\Delta t\right)/z_{t}^{V_{t}}+1} \end{array}\right] \end{array}.$$

Given that $u_{t}^{V}\omega_{y}^{t}\Delta t-v_{t}^{V}\omega_{x}^{t}\Delta t+\left(t_{x}\omega_{y}^{t}\Delta t-t_{y}\omega_{x}^{t}\Delta t\right)/z_{t}^{V_{t}}\ll 1$ in this study, we have:

(24) $$\begin{array}{ll} \left[\begin{array}{c} u_{t+\Delta t}^{V}\\ v_{t+\Delta t}^{V} \end{array}\right] & \approx \left[\begin{array}{c} u_{t}^{V}+v_{t}^{V}\omega_{z}^{t}\Delta t-\omega_{y}^{t}\Delta t+\left(t_{y}\omega_{z}^{t}\Delta t-\omega_{y}^{t}t_{z}\Delta t\right)/z_{t}^{V_{t}}\\ -u_{t}^{V}\omega_{z}^{t}\Delta t+v_{t}^{V}+\omega_{x}^{t}\Delta t+\left(-t_{x}\omega_{z}^{t}\Delta t+t_{z}\omega_{x}^{t}\Delta t\right)/z_{t}^{V_{t}} \end{array}\right]\\ & =\left[\begin{array}{c} u_{t}^{V}\\ v_{t}^{V} \end{array}\right]+\left[\begin{array}{c} v_{t}^{V}\omega_{z}^{t}-\omega_{y}^{t}+\left(t_{y}\omega_{z}^{t}-\omega_{y}^{t}t_{z}\right)/z_{t}^{V_{t}}\\ -u_{t}^{V}\omega_{z}^{t}+\omega_{x}^{t}+\left(-t_{x}\omega_{z}^{t}+t_{z}\omega_{x}^{t}\right)/z_{t}^{V_{t}} \end{array}\right]\Delta t\\ & =\left[\begin{array}{c} u_{t}^{V}\\ v_{t}^{V} \end{array}\right]+\left[\begin{array}{c} \lambda \\ \xi \end{array}\right]\Delta t \end{array}.$$

Moreover, given that $M=\left[\begin{array}{ccc} \alpha_{x} & & u_{0}\\ & \alpha_{y} & v_{0}\\ & & 1 \end{array}\right]$, we have

(25) $$\left[\begin{array}{c} u_{t}\\ v_{t}\\ 1 \end{array}\right]=\left[\begin{array}{ccc} \alpha_{x} & & u_{0}\\ & \alpha_{y} & v_{0}\\ & & 1 \end{array}\right]\left[\begin{array}{c} u_{t}^{V}\\ v_{t}^{V}\\ 1 \end{array}\right]=\left[\begin{array}{c} \alpha_{x}u_{t}^{V}+u_{0}\\ \alpha_{y}v_{t}^{V}+v_{0}\\ 1 \end{array}\right]\;\mathrm{and}$$

(26) $$\left[\begin{array}{c} u_{t+\Delta t}\\ v_{t+\Delta t}\\ 1 \end{array}\right]=\left[\begin{array}{ccc} \alpha_{x} & & u_{0}\\ & \alpha_{y} & v_{0}\\ & & 1 \end{array}\right]\left[\begin{array}{c} u_{t+\Delta t}^{V}\\ v_{t+\Delta t}^{V}\\ 1 \end{array}\right]=\left[\begin{array}{c} \alpha_{x}u_{t+\Delta t}^{V}+u_{0}\\ \alpha_{y}v_{t+\Delta t}^{V}+v_{0}\\ 1 \end{array}\right]=\left[\begin{array}{c} \alpha_{x}u_{t}^{V}+u_{0}+\lambda \alpha_{x}\Delta t\\ \alpha_{y}v_{t}^{V}+v_{0}+\xi \alpha_{y}\Delta t\\ 1 \end{array}\right]=\left[\begin{array}{c} u_{t}\\ v_{t}\\ 1 \end{array}\right]+\left[\begin{array}{c} \lambda \alpha_{x}\Delta t\\ \xi \alpha_{y}\Delta t\\ 1 \end{array}\right].$$

When Δt=−τ/2, we can modify the coordinates of the checkerboard corners, thereby eliminating the smearing effect.

The image process algorithm is summarized as Table 1.

The corner extraction accuracy can be significantly enhanced by using the above checkerboard corner detection algorithm, especially when a smearing effect exists. 

### 2.4. Description of the Estimator

There are many methods that can solve the equations that provide the extrinsic parameters, such as EKF [33,34], genetic algorithm [35], and so on. Here, we utilize an EKF for calibrating the extrinsic parameters. The EKF algorithm is briefly introduced in Table 2. The state vector x includes the extrinsic parameters (translation vectors $t_{BI}^{I}$, $t_{BV}^{V}$ and Euler angle $\psi_{B}^{I}$, $\psi_{B}^{V}$), the IMU bias ($b_{a}$ and $b_{g}$), the camera initial parameter ($t_{FV_{0}}^{V_{0}}$ and $\psi_{F}^{V_{0}}$), and the rotation angle ($\delta \beta_{R}$). Meanwhile, the measurement vector includes the accelerometer ($f_{x}^{I}$ and $f_{y}^{I}$) and gyro ($\omega_{x}^{I}$ and $\omega_{y}^{I}$) measurements as well as the extracted checkerboard corners ($u_{x}^{}$,$v_{x}^{}$, $u_{y}^{}$, and $v_{y}^{}$). 

## 3. Simulation and Real-World Experiment

### 3.1. Simulation

A simulation test is designed to validate the performance of the extrinsic parameter calibration method. During the simulation, the turntable is assumed to demonstrate swinging motions around the *x* and *y* axes. The swinging rule is $\omega_{k}=A_{k}\sin\left(2\pi f_{k}+\varphi_{k}\right)+\omega_{k0}$, where k=x,y, $\omega_{k}$ denotes the angular velocity, $A_{k}$ and $f_{k}^{}$ denote the swinging amplitude and frequency, respectively, and $\varphi_{k}$ and $\omega_{k0}$ denote the initial phase and swinging center, respectively. The swinging parameters of the simulation are defined in Table 3.

The true values for simulation data of the visual/inertial integrated system are defined in Table 4.

Given the parameters in Table 3 and Table 4, the true measurement of the gyroscope and accelerometer can be simulated by using the dynamics equation. The true measurement of the camera (pixel coordinates of the checkerboard corners) can be simulated by using the image model. When the errors in Table 5 are added into the ideal measurement data, real inertial sensor and camera outputs can be generated. The sampling rates of the IMU and camera are 100 Hz and 10 Hz, respectively.

**Figure 4:**
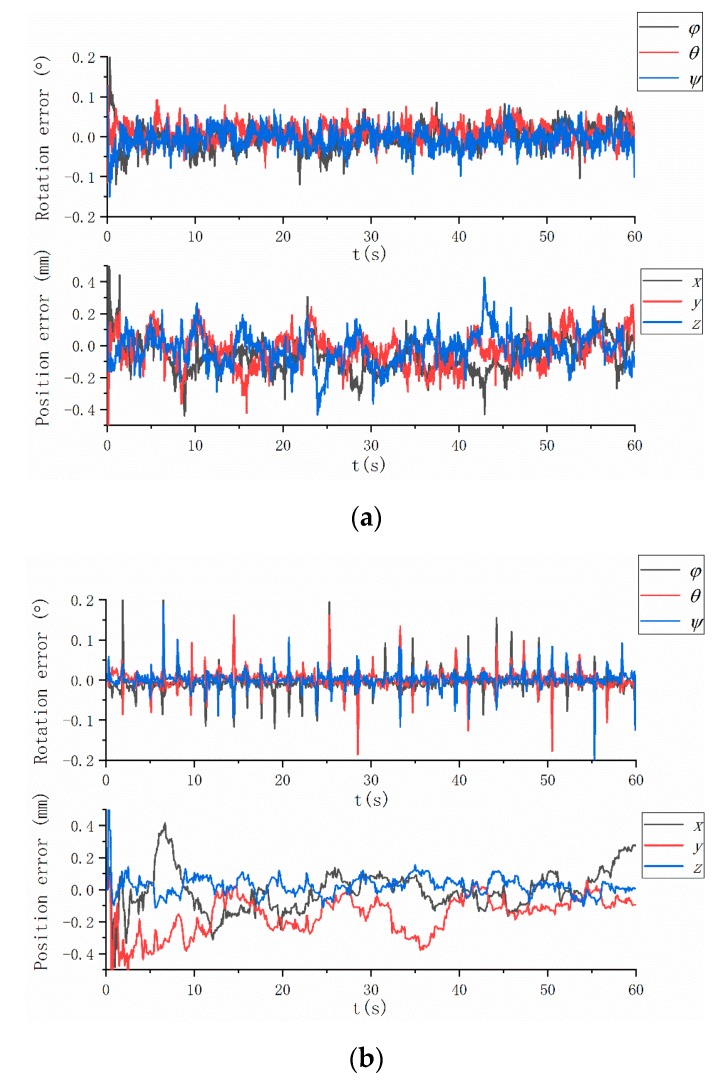
Simulation results: (**a**) rotation and position errors of MIMU, and (**b**) rotation and position errors of the camera.

The alignment errors are shown in Figure 4, whereas the alignment error statistics are listed in Table 6. The calibration method can evaluate the extrinsic parameters correctly. The attitude error is <0.03° for each Euler angle, and the position error is <0.10 mm for each position vector component. 

### 3.2. Real-World Experiment

#### 3.2.1. Experiment Setting

**Figure 5:**
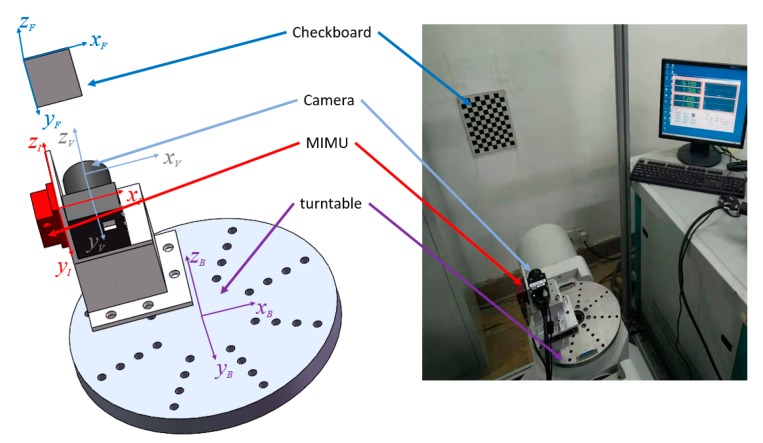
Calibration architecture.

A calibration experiment is conducted to confirm the validity of the proposed method and to evaluate the accuracy of the system. Figure 5 shows the experiment architecture, whereas Table 7 presents the main devices. The system body frame coincides with the turntable frame for the sake of simplicity because the mechanical interface of the turntable frame is clearly defined.

The experiment is designed as follows. First, frames {*I*_0_}, {*V*_0_}, and {*B*_0_} are defined to coincide at the initialization time. The system body, camera, and MIMU coordinates are fixed with the turntable, camera, and MIMU, respectively. Second, the turntable is manually controlled to rotate around its axes, whereas the visual/inertial integrated system moves along with the turntable. 

The intrinsic parameters obtained through Zhang’s method [27] are shown in Table 8. The calibration achieved an accuracy of 0.08 pixel based on 18 images.

#### 3.2.2. Experiment Results and Discussion

**Figure 6:**
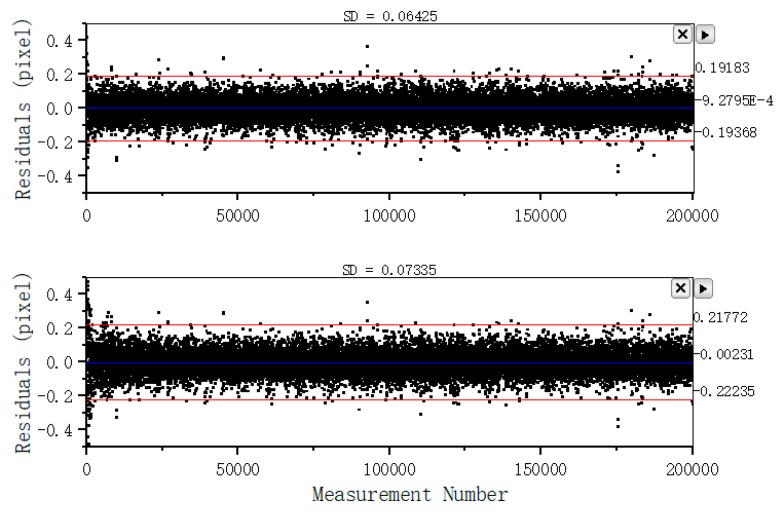
Measurement residuals with their 3σ bounds. The top and bottom images present the results for methods before and after modification, respectively.

The test results are presented in this section. The extrinsic calibration results before and after the checkerboard corner modification are nearly similar. The standard uncertainty of the modified method (0.15 mm) (Table 10) is lower than that of the unmodified method (0.18 mm) (Table 9). The camera measurement residuals of the two methods are different as shown in Figure 6. The measurement residuals’ 3σ bound of the method based on a linear constraint is approximately 0.193 pixels, whereas the measurement residuals’ 3σ bound of the unmodified method is approximately 0.220 pixels. Thus, the motion blur correction described in Section 2.3 is effective.

The difference of IMU calibration parameters before and after modification is not significant. The camera measurements can suppress the angle integral error due to IMU’s drift, and increase the accuracy of IMU extrinsic parameters calibration results. Thus, more accuracy camera measurements will lead to more accuracy IMU extrinsic parameters calibration results. But the accuracy of IMU extrinsic parameters calibration results is closed before and after checkerboard corner modification, through compare the results in Table 9 and Table 10. There may be three reasons explain it.

1. For camera measurements, the rotation angle is computed based on all the checkerboard corners’ pixel coordinates. The effects of motion blur are eliminate by involve all corners into computation process.
2. The calibration process is not long, so the effect of accumulation error is not significant.
3. There are system errors exits, such as the non-orthogonal of rotation axes, and the time delay of data acquisition, which also influence the error level.

**Figure 7:**
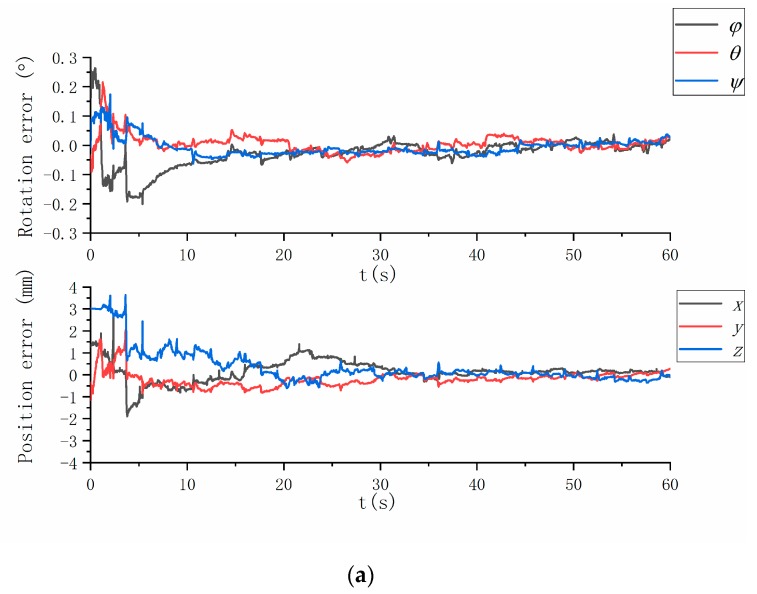

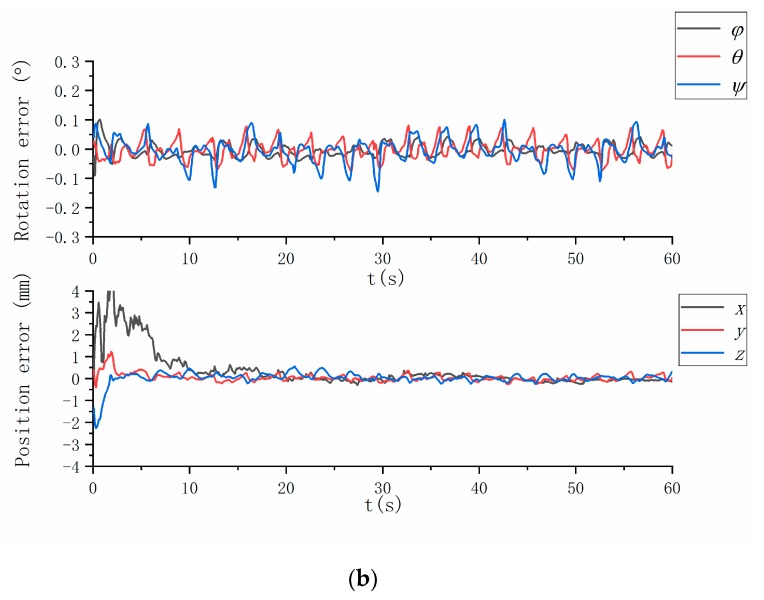
Results of the calibration experiment: (**a**) attitude and position errors of MIMU and (**b**) camera.

Table 10 and Figure 7 summarize the experiment results. The origin of the MIMU frame is discussed in the sbg-IMU user manual, while that of the camera is the optical center of the lens. Therefore, the translation vector between the MIMU and camera can be roughly evaluated. The results that are evaluated based on the mechanical structure coincide with those that are evaluated by using EKF.

The experiment results, the existing problems and possible reasons, the strategies for improving the results, and some directions for future work are presented below:

1. IMU and camera frames are aligned to the system body frame. The standard deviations of the three-axis position error are (0.13, 0.15, 0.11) mm and (0.12, 0.10, 0.13) mm for the MIMU and camera, respectively. Meanwhile, the standard deviations of the three-axis Euler angle error are (0.02°, 0.02°, 0.02°) and (0.03°, 0.04°, 0.02°) for the MIMU and camera, respectively. Compare the difference between the method with corner correction and without corner correction. We find the camera extrinsic parameters’ accuracy of former is higher than the latter (Table 9 and Table 10), and the camera measurement residuals of former is lower than the latter (Figure 6). It indicates the corner correction described in Section 2.3 is effective. The reasons why the difference of IMU calibration parameters before and after modification is not significant have been briefly discussed.
2. There are three errors affecting the calibration accuracy. Firstly, the calibration errors of IMU and camera intrinsic parameters, which affect the measurements’ accuracy. At present, a reasonable choice of camera calibration method ensures that imaging accuracy reaches the sub-pixel level, and IMU is factory calibrated. Secondly, the time delay between the IMU and camera data acquisition, which affects the calibration accuracy and stability of filter. We align the data by the time label (both IMU and camera data are marked on the time label, respectively), and don’t evaluate the time delay exactly. Thirdly, we ignore the non-orthogonality of the turntable axes, and the turntable has been factory calibrated. Further research on a solution without orthogonal axes could be performed.
3. We can observe the convergence of each parameter in the EKF process. The experiment results show that the method is valid and is not restricted in the Kalman filter. Some optimal algorithms, such as the particle filter and Levenberg–Marquardt algorithm can also be used. The calibration parameters are obtained, and the complete visual/inertial integrated system is established. Future research may focus on the calibration in the navigation process, and the proposed method may be seen as a standard calibration step in factory production and user operation.

## 4. Conclusions

An extrinsic parameter calibration method for a visual/inertial integrated system is developed based on a swinging motion. A checkerboard corner detection algorithm is then utilized to detect checkerboard corners with a smearing effect. The extrinsic parameter calibration method is developed based on the imaging model and dynamic equation. This method is validated by performing a simulation and a real-world experiment, which results highlight the effectiveness of the proposed method. This method can also be seen as a standard calibration step and used for visual/inertial systems, especially for visual and inertial navigation integrated systems.

## Figures and Tables

**Table 1:** Checkerboard Corner Detection under Motion Blur.

Inputs: Camera and IMU measurements
Output: Coordinates of checkerboard corners
Algorithm:
1. Deblur the image with the aid of inertial data.
2. Roughly extract the checkerboard corners based on the equation of the second gradient.
3. Refine the corners through the linear constraint.
4. Modify the checkerboard corners with IMU-aided.

**Table 2:** EKF updating process.

**1. State Equation**
$x=\left[\begin{array}{c} x_{I}\\ x_{V} \end{array}\right]$, $x_{I}={\left[\begin{array}{cccc} t_{BI}^{I} & \psi_{B}^{I} & b_{a} & b_{g} \end{array}\right]}^{T}$, $x_{V}={\left[\begin{array}{ccccc} t_{BV}^{V} & \psi_{B}^{V} & t_{FV_{0}}^{V_{0}} & \psi_{F}^{V_{0}} & \delta \beta_{R} \end{array}\right]}^{T}$
**2. Measurement Model**
$z=\left[\begin{array}{c} z_{I}\\ z_{V} \end{array}\right]$, $z_{I}={\left[\begin{array}{cccc} f_{x}^{I} & \omega_{x}^{I} & f_{y}^{I} & \omega_{y}^{I} \end{array}\right]}^{T}$, $z={\left[\begin{array}{cccc} u_{x}^{} & v_{x}^{} & u_{y}^{} & v_{y}^{} \end{array}\right]}^{T}$
**3. Updating**
${\hat{x}}_{j}^{-}=\Phi_{j-1}^{}{\hat{x}}_{j}^{+},\;P_{j}^{-}=\Phi_{j-1}^{}P_{j-1}^{+}\Phi_{j-1}^{T}+Q_{j-1}^{},\;\Phi =\left[\begin{array}{cc} \Phi_{I} & 0_{12,14}\\ 0_{14,12} & \Phi_{V} \end{array}\right],\;Q=\left[\begin{array}{cc} Q_{I} & 0_{12,14}\\ 0_{14,12} & Q_{V} \end{array}\right],\;K_{j}^{}=P_{j}^{-}H_{j}^{}{\left(H_{j}^{}P_{j}^{-}H_{j}^{T}+R_{j}^{}\right)}^{-1},\;{\hat{x}}_{j}^{+}=x_{j}^{-}+K_{j}^{}\delta z_{j}^{},\;P_{j}^{+}=\left(I-K_{j}^{}H_{j}^{}\right)P_{j}^{-},\;P=\left[\begin{array}{cc} P_{I} & P_{IV}\\ P_{IV}^{T} & P_{V} \end{array}\right]$

**Table 3:** Swinging parameters.

Rotation Axis	X Axis	Y Axis
Amplitude (deg/s)	50	10
Frequency (Hz)	2	3
Initial phase (°)	90	90
Swinging center (deg/s)	0	0

**Table 4:** The true values for simulation data.

Parameters	Value
Euler angle $\psi_{B}^{I}$ (°)	[−0.57, 0.57, 0.57]
Translation $t_{BI}^{B}$ (m)	[0.05, −0.05, 0.05]
Euler angle $\psi_{B}^{V}$ (°)	[0.57, −0.57, 0.57]
Translation $t_{BV}^{B}$ (m)	[0.10, 0.10, 0.10]
Euler angle $\psi_{F}^{V_{0}}$ (°)	[5.73, 5.73, 5.73]
Translation $t_{F}^{V_{0}}$ (m)	[0.10, 0.10, 1.10]
Gravity accelerometer $g_{}^{I_{0}}$ (m/s^2^)	[0.10, 9.70, 0.10]

**Table 5:** Sensor errors.

	Gyro Noise (deg/h)		Accelerometer Noise (µg)	
	Constant	Random	Constant	Random
*x* axis	0	20	0	200
*y* axis	0	20	0	200
*z* axis	0	20	0	200
	**Camera Noise (Pixel)**			
	**Constant**		**Random**	
*u* axis	0		1	
*v* axis	0		1	

**Table 6:** Results and deviation of simulation.

Rotation Error ^1^				Position Error			
Matrix	Euler Angle	Mean (°)	Std ^2^ (°)	Vector	Component	Mean (mm)	Std (mm)
$C_{B}^{I}$	$\phi_{I}$	0.0011	0.0236	$t_{BI}^{B}$	$t_{BI,x}^{B}$	−0.0462	0.1048
	$\theta_{I}$	0.0010	0.0213		$t_{BI,y}^{B}$	−0.0108	0.0907
	$\psi_{I}$	−0.0003	0.0223		$t_{BI,z}^{B}$	0.0219	0.0922
$C_{B}^{V}$	$\phi_{V}$	−0.0022	0.0256	$t_{BV}^{B}$	$t_{BV,x}^{B}$	0.0183	0.0993
	$\theta_{V}$	0.0008	0.0253		$t_{BV,y}^{B}$	−0.1221	0.0956
	$\psi_{V}$	0.0016	0.0253		$t_{BV,z}^{B}$	0.0244	0.0690

^1^ The error is the different between the simulation results and the true value. ^2^ Std is the standard deviation.

**Table 7:** Major devices involved in the experiment.

Device	Manufacturer	Model	Main Parameters
Camera	Dalsa Image, Canada	FA-21-1M120	Resolution: 1024 × 1024 Refresh rate: 10 frames/s Lens respective scale factor: 1600; FOV ~45°
MIMU	SBG Systems, France	Ellipse-A2-G4	Accelerometer: 8 g full scale, 20 µg in-run bias Instability; Gyro: 450 deg/s full scale, 8 deg/h in-run bias instability
Turntable	Aircraft Industry Precision Engineering Institute, China	902-1	Accuracy of angular position: 8′’ in both axes

**Table 8:** Calibration result of intrinsic parameters of camera.

Parameter	$\alpha_{x}$	$\alpha_{y}$	$u_{0}$	$v_{0}$	γ	*k*_1_	*k*_2_	*P*_1_	*P*_2_^1^
Calibration result	1123.0114	1123.9004	527.7581	527.8911	0	−0.1027	0.1738	0.0012	0.0014
error	0.4994	0.4916	0.5249	0.6548	0	0.0008	0.0035	0.0001	0.0001

^1^*k*_1_ and *k*_2_ are the radial distortion of the lens, while *P*_1_ and *P*_2_ are the tangential distortion.

**Table 9:** Results and deviation of the experiment based on the unmodified corner detection method.

Rotation Matrix				Position Vector			
Matrix	Euler Angle	Calibration Result (°)	Error ^1^ 1σ (°)	Vector	Component	Calibration Result (mm)	Error 1σ (mm)
$C_{B}^{I}$	$\phi_{I}$	−0.537	0.0166	$t_{BI}^{B}$	$t_{BI,x}^{B}$	−102.781	0.133
	$\theta_{I}$	−2.183	0.0168		$t_{BI,y}^{B}$	−246.875	0.145
	$\psi_{I}$	0.480	0.0185		$t_{BI,z}^{B}$	−1.362	0.108
$C_{B}^{V}$	$\phi_{V}$	−1.176	0.0372	$t_{BV}^{B}$	$t_{BV,x}^{B}$	−77.434	0.147
	$\theta_{V}$	0.085	0.0488		$t_{BV,y}^{B}$	−229.920	0.113
	$\psi_{V}$	−0.175	0.0178		$t_{BV,z}^{B}$	39.264	0.184

^1^ The error is evaluated via the standard deviation (the same below).

**Table 10:** Results and deviation of the experiment based on the modified corner detection method.

Rotation Matrix				Position Vector			
Matrix	Euler Angle	Calibration Result (°)	Error 1σ (°)	Vector	Component	Calibration Result (mm)	Error 1σ (mm)
$C_{B}^{I}$	$\phi_{I}$	−0.536	0.0163	$t_{BI}^{B}$	$t_{BI,x}^{B}$	−102.752	0.128
	$\theta_{I}$	−2.183	0.0165		$t_{BI,y}^{B}$	−246.966	0.146
	$\psi_{I}$	0.480	0.0186		$t_{BI,z}^{B}$	−1.321	0.107
$C_{B}^{V}$	$\phi_{V}$	−1.197	0.0320	$t_{BV}^{B}$	$t_{BV,x}^{B}$	−77.091	0.117
	$\theta_{V}$	0.085	0.0376		$t_{BV,y}^{B}$	−229.742	0.103
	$\psi_{V}$	−0.187	0.0205		$t_{BV,z}^{B}$	38.294	0.128
